# Validity and reproducibility of the PERSIAN Cohort food frequency questionnaire: assessment of major dietary patterns

**DOI:** 10.1186/s12937-024-00938-0

**Published:** 2024-03-13

**Authors:** Sareh Eghtesad, Sahar Masoudi, Maryam Sharafkhah, Bahram Rashidkhani, Ali Esmaeili-Nadimi, Farid Najafi, Elnaz Faramarzi, Reza Homayounfar, Pedram Ebrahimnejad, Alireza Ansari-Moghaddam, Mohammadreza Mirjalili, Hossein Poustchi, Walter C Willett, Reza Malekzadeh, Azita Hekmatdoost

**Affiliations:** 1 Liver and Pancreatobiliary Diseases Research Center, Digestive Diseases Research Institute, Tehran University of Medical Sciences, Tehran, Iran; 2 Digestive Diseases Research Center, Digestive Diseases Research Institute, Tehran University of Medical Sciences, Tehran, Iran; 3 Department of Community Nutrition, National Nutrition & Food Technology Research Institute, Shahid Beheshti University of Medical Sciences, Tehran, Iran; 4 Non-Communicable Diseases Research Center, Rafsanjan University of Medical Sciences, Rafsanjan, Iran; 5 Research Center for Environmental Determinants of Health (RCEDH), Nutritional Sciences Department, Kermanshah University of Medical Sciences, Kermanshah, Iran; 6 Liver and Gastrointestinal Diseases Research Center, Tabriz University of Medical Sciences, Tabriz, Iran; 7 Noncommunicable Diseases Research Center, Fasa University of Medical Sciences, Fasa, Iran; 8 Faculty of Nutrition and Food Technology, National Nutrition and Food Technology Research Institute, Shahid Beheshti University of Medical Sciences, Tehran, Iran; 9 Pharmaceutical Sciences Research Center, Hemoglobinopathy Institute, Mazandaran University of Medical Sciences, Sari, Iran; 10 Department of Pharmaceutics, Faculty of Pharmacy, Mazandaran University of Medical Sciences, Sari, Iran; 11 Health Promotion Research Center, Zahedan University of Medical Sciences, Zahedan, Iran; 12 Department of Intensive Care, School of Medicine, Shahid Sadoughi University of Medical Sciences, Yazd, Iran; 13 Department of Nutrition, Harvard T.H. School of Public Health, Boston, MA USA; 14 Department of Epidemiology, Harvard T.H. School of Public Health, Boston, MA USA; 15 Department of Clinical Nutrition & Dietetics, National Nutrition & Food Technology Research Institute, Shahid Beheshti University of Medical Sciences, No.7, West Arghavan St. Farahzadi Blvd., Tehran, 19816-19573 Iran

**Keywords:** Food frequency questionnaire, Dietary patterns, PERSIAN Cohort, Validity, Reproducibility

## Abstract

**Background:**

Dietary patterns, encompassing an overall view of individuals’ dietary intake, are suggested as a suitable means of assessing nutrition’s role in chronic disease development. The aim of this study was to evaluate the validity and reproducibility of a food frequency questionnaire (FFQ) designed for use in the Prospective Epidemiological Research Studies in IrAN (PERSIAN), by comparing major dietary patterns assessed by the FFQ with a reference method.

**Methods:**

Study participants included men and women who enrolled in the PERSIAN Cohort Study at seven of the eighteen centers. These centers were chosen to include dietary variations observed among the different Iranian ethnic populations. Two FFQ were completed for each participant over a one-year study period (FFQ1 upon enrollment and FFQ2 at the end of the study), with 24 interviewer-administered 24-hour dietary recalls (24 h) being completed monthly in between. Spearman correlation coefficients (SCC) were used comparing FFQs 1 and 2 to the 24 h to assess validity, while FFQ1 was compared to FFQ2 to assess reproducibility of the questionnaire.

**Results:**

Three major dietary patterns—Healthy, Low Protein/High Carb and Unhealthy—were identified, accounting for 70% of variance in the study population. Corrected SCC ranged from 0.31 to 0.61 in the validity and from 0.34 to 0.57 in reproducibility analyses, with the first two patterns, which accounted for over 50% of population variance, correlated at above 0.5 in both parameters, showing acceptable findings.

**Conclusions:**

The PERSIAN Cohort FFQ is suitable for identification of major dietary patterns in the populations it is used for, in order to assess diet-disease relationships.

**Supplementary Information:**

The online version contains supplementary material available at 10.1186/s12937-024-00938-0.

## Background

What an individual eats throughout their lifetime affects their health and disease development; whether to study dietary patterns, foods or nutrients to best assess this relationship, has been debated over the years. Nutrients and components of foods were the primary focus of nutritional epidemiology in the past because many nutrients and their required levels to maintain health were unknown, thus nutrient deficiencies were commonly seen, especially in developing countries with more limited access to a variety of foods [1–4]. Today however, chronic diseases are on the rise globally, following the obesity pandemic, and nutrition’s role in disease development has taken a different turn. While the etiology of chronic diseases is multifactorial, nutrition continues to have an important effect; however, not through single nutrients or foods, which are rarely the cause of chronic diseases. Instead, the overall dietary composition throughout an individual’s lifetime has become the focus and therefore, dietary patterns have emerged as a suitable means of evaluating nutrition in chronic diseases [2, 4, 5].

The relationship between dietary patterns and chronic diseases can be well-examined in longitudinal cohort studies [6, 7]. The largest cohort study in Iran—the Prospective Epidemiological Research Studies in IrAN (PERSIAN)—studying risk factors associated with common chronic diseases, has gathered detailed baseline information on many exposures, including diet, through a food frequency questionnaire (FFQ) [8, 9]. While the validation of the PERSIAN Cohort FFQ has been previously evaluated at the food group level, assessing these parameters at the level of dietary patterns is also desired to ensure that data collected by this questionnaire can adequately depict future findings regarding diet and diseases [10]. The aim of this study, therefore, is to assess the validity and reproducibility of major dietary patterns identified by the PERSIAN Cohort FFQ by comparison to 24-hour dietary recalls (24 h).

## Methods

### PERSIAN Cohort study

The PERSIAN Cohort Study is a closed-enrollment population-based prospective cohort including 163,770 men and women 35–70 years of age from 18 geographically distinct areas of Iran. Baseline data collection took place from 2015 to 2020, where questionnaires regarding various lifestyle and environmental exposures as well as pertinent medical histories were completed for all participants, including a food frequency questionnaire for the assessment of nutrition-disease relationships. Individuals are followed yearly to assess the study endpoints of major non-communicable disease (NCD) development and/or death. The main goal of the PERSIAN Cohort is to study the incidence and burden of major NCDs as well as the risk factors associated with them. The rationale, design and objectives of the PERSIAN Cohort Study have been detailed previously [8, 9].

### Study population and data collection

Participants of this validation study include cohort enrollees from seven of the eighteen PERSIAN Cohort centers. We chose the Fasa, Rafsanjan, Azar, Yazd, Ravansar, Zahedan, and Tabari centers, to include adequate dietary variations from major ethnic populations and geographical areas of Iran. Participant recruitment for this study, relied on enrollment in the pilot phase of the main cohort and 1,260 individuals (180 from each of the seven centers) who had enrolled in the cohort, were also invited to participate in this study. Those who agreed (*n* = 1097, 87%), completed two FFQs (FFQ1 completed at the start and FFQ2 completed at the end of the one-year study duration), as well as twenty-four 24 h in between. Individuals missing > 12 or all 24 h in one season were excluded from the study, leaving 978 participants in the validity assessment comparing FFQ1 vs. 24 h. Those missing FFQ2 were also excluded from any analysis requiring data from this questionnaire, leaving 891 for comparing FFQ2 vs. 24 h (validity assessment) as well as the reproducibility analysis comparing FFQ1 to FFQ2. All participants signed a written informed consent to participate in this study, which was approved by the ethics committee of the Digestive Diseases Research Institute, Tehran University of Medical Sciences (IR.TUMS.DDRI.REC.1398.001).

### Dietary assessment

#### PERSIAN Cohort FFQ

The PERSIAN Cohort FFQ includes 113 food items in the following 9 food categories: Breads and Grains, Legumes, Dairy, Meats and Meat Products, Vegetables, Fruits, Fats/Oils and Nuts, Sweets, and Miscellaneous and inquires about their usual intake over the year prior to its completion. These items were chosen by expert dietitians, from two previously validated FFQs in Iran [the Golestan Cohort Study (GCS) FFQ including 150 food items [11] and the Tehran Lipid and Glucose Study (TLGS) FFQ including 165 [12]], with an attempt to design a comprehensive yet shorter FFQ, since about 17 questionnaires were completed for PERSIAN Cohort participants upon enrollment, and a shorter FFQ was desired to reduce participant fatigue, in turn affecting response accuracy. In addition to the 113 items, about 5–10 items were also added to each cohort center’s FFQ, including major energy-contributing or nutrient-dense local foods that were consumed on a regular basis in that cohort population, mostly consisting of local breads and sweets. After data collection, these local items were equated to the standard FFQ food items, based on their major ingredients or recipes, then used in analyses.

The PERSIAN Cohort FFQ was interviewer-administered with the interviewers at all cohort centers being trained by the same individual following the same protocols and techniques. Participants were questioned about their frequency of intake for each food item over the year prior to their interview, reported as daily, weekly, monthly or yearly consumption, then asked about the portion size they usually ate each time, based on pre-defined portion sizes for each item. To increase the accuracy of portion size reporting, cups, dishes and utensils, as well as a food album picturing the standard portions were used at the time of the interviewing [13]. Using the reported intakes for frequency and portion size, daily intake of each item was calculated in grams (grams/day). For example, if an individual reported consuming apples 5 time/week, each time eating 1 medium-sized apple, the frequency was converted to 0.71 times per day, which was then multiplied by the weight of the standard apple to acquire the grams of apple consumed each day. Further details about the questionnaire design and administration have been previously described [10].

#### Reference methods

During the one-year interval between FFQ1 and FFQ2 completion, two 24 h were also completed for each participant, every month (for a total of 24 recalls). The 24 h were completed in-person and by the same trained interviewers who completed the FFQs. In instances when it was not possible for a participant to attend the cohort center, the 24 h was completed over the phone. The United States Department of Agriculture (USDA) multiple-pass method was used to complete the 24 h [14]. For the sake of data analysis, foods reported in the 24 h were matched to food items included in the FFQ. For over 95% of the food items, an exact match was possible and the remaining were matched to the nearest FFQ food item(s) based on their major macro- and micro-nutrient contents.

### Food grouping

We grouped the FFQ food items for a simpler dietary pattern analysis. The same food grouping used in the food group validation study of the PERSIAN Cohort FFQ was used in this study as well [10]. The nine food categories in the FFQ, which were based on the USDA MyPlate food groups, were used as the basis of the food grouping [15]. Then, the nutrient content of items within each category were evaluated and items with similar nutrients or with specific distinct nutrients were grouped together, for a total of 23 food/food groups (Table 1).

**Table 1:** Food grouping of the PERSIAN Cohort FFQ items

Whole Grain	Sangak Bread, Wheat/Oats/Barley, Kornoo Bread*, Barley Bread/Diabetic Breads, Zahedani Taftoon Bread*
White Grain	Lavash Bread, Barbari Bread, Baguettes, Rice, Pasta/Noodles, Tiri Bread*, Kalaneh*, Traditional Breads*, Burak Bread*, Tanoori Bread*, Dried Bread*
Beans	Beans, Chickpeas, Mung beans/Lentil/Dhal, Split peas, Soybeans, Fava/Lima beans
Fish	Fish (all types), Canned tuna
Red Meat	Red meat (including lamb, goat, mutton, beef), Camel meat*
Processed Meat	Sausages/Kilbasa/Salami (all types)
Chicken	Chicken, Other poultry (duck, goose, ….)
Egg	Eggs
Cheese	Cheese (all types)
Dairy	Milk, Yogurt, Doogh (Ayran), Kashk (Whey), Flavored Milk (chocolate, strawberry, banana, etc.), Yellow Kashk*, Colostrum*
Vegetables	Lettuce, Cabbage, Tomatoes, Cucumbers, Fresh leafy greens, Cooked leafy greens, Eggplant, Celery, Beets/Turnips, Potatoes, Carrots, Garlic, Onions, Bell Peppers, Mushrooms, Corn, Green peas, Green beans, Zucchini, Green Peppers (Jalopeno), Okra*, Local Green Herbs*
Juice	Fresh Fruit Juice
Fruit	Cantaloup Melon, Honeydew, Watermelon, Apricot, Sweet/Sour Cherries, Nectarines, Peaches, Prunus, Fresh Berries, Strawberries, Plums, Fresh Figs, Grapes, Pears, Apples, Kiwi, Citrus Fruits (including Oranges, Tangerines, Lemon, Lime, Grapefruit), Pomegranate, Bananas, Persimmon, Mango*, Olive Fruit*, Jujube*, Sapodilla*, Pineapples*
Dried Fruit	Dates, Dried fruit, Raisins
Solid oil	Margarine, Butter, Hydrogenated oils/Animal Fats, Cream/Clotted Cream, Kermanshahi Ghee*
Liquid Oil	Vegetable Oils (all types excluding olive oil)
Olive/ Olive Oil	Olives, Olive oil
Nuts	Walnuts, Peanuts, Other nuts (including pistachios, almonds, cashews, etc.), Seeds (sunflower, zucchini, watermelon), Chilgoza Pine*
Sugar	Sugar Cubes, Nabbat (Crystalized sugar), Honey, Jams, Syrups (all types), Sugar, Red Sugar/Sugar Beet*
Sweets	Cookies/Dry sweets and cakes (i.e. pound cakes), Creamed/Puffed sweets and cakes, Ice cream, Chocolate, Tahini (all types), Noughat*, Masghati*, Komache-Sen*, Zaboli Cookies*
Sweet Drinks	Soft drinks, Non-alcoholic Beer, Juice (all types except for fresh)
Tea	Tea
Salt	Salt, Dalal*

Items that are listed on the FFQ are separated by commas (,). Those separated by slashes (/) were asked as one item. Items designated with an asterix (*) were local items that were equated to the standard items based on their major ingredients/recipes

### Statistical analysis

Means and standard deviations (mean ± SD) as well as mean difference and 95% confidence intervals (95% CI) of the food group intakes were calculated from the 24 h and the two FFQs. Principal Component Analysis (PCA) was used to derive food patterns based on energy-adjusted intakes of the 23 food groups from each questionnaire. Food groups were energy-adjusted by the nutrient-density approach [16]. Eigenvalues > 1, as well as the elbow of scree plots (supplementary Fig. 1) were considered when deciding on the number of factors to retain.

The relative validity and reproducibility of dietary patterns derived from the FFQ were examined by calculating Spearman rank correlation coefficients (SCC) and 95% CI between diet pattern scores obtained from FFQs 1 and 2 vs. the 24 h (relative validity), and FFQ1 vs. FFQ2 (reproducibility).

Corrected correlations were also calculated, taking into account random within-person errors, using the following formula:

$${r}_{t}={r}_{0}\sqrt{\left(1+\raisebox{1ex}{$\lambda $}\!\left/ \!\raisebox{-1ex}{$k$}\right.\right)}$$

where ${r}_{t}$ is the corrected correlation between the dietary pattern scores derived from the FFQs and 24 h, ${r}_{0}$ is the observed correlation, $\lambda$ is the ratio of estimated within-person and between-person variation in dietary pattern scores derived from the 24 h, and k is the number of repeated observations of the dietary recalls (k = 24) [17]. Agreement between dietary pattern scores across the recorded intakes was determined using the Bland and Altman method (supplementary materials 1) [18, 19]. All statistical analyses were performed using the statistical software STATA 12 (StataCorp, College Station, TX, USA).

## Results

We included 978 men and women in our study. Mean age of participants was 46.6 ± 8.25 and 58% of participants were female. About 43% of the population were illiterate or with only primary education, while 13% had a university degree. Individuals from both urban and rural areas were included, with the majority (81.2%) residing in urban areas, parallel to the entire PERSIAN Cohort population.

Mean intake of each food group recorded by FFQ1, 2 and the 24 h are shown in Table 2, ranging from 0.42 ± 0.42 to 570 ± 303 in FFQ1, 0.48 ± 0.51 to 559 ± 308 in FFQ2, and 0.38 ± 0.17 to 500 ± 233 in the 24 h. The highest and lowest mean intakes belonged to *tea* and *olive/olive oil*, respectively, in all three questionnaires. Based on the mean differences obtained between the FFQs and the 24 h, the FFQs tend to overestimate *white grains, processed meat, dairy, vegetables, fruit, dried fruit, solid* and *liquid oils, nuts, sugars, sweet drinks, tea* and *salt* consumption both times completed, while *fish, red meat, chicken* and *sweets* intake were underestimated by both questionnaires.

**Table 2:** Mean ± SD of food groups in each questionnaire (g/day) as well as mean differences (95%CI) between FFQ1 vs. 24 h and FFQ2 vs. 24 h

Food Groups	Mean ± SD			Mean Difference (95%CI)	
	FFQ1	FFQ2	24 h	FFQ1 vs. 24 h	FFQ2 vs. 24 h
Whole Grain	24.6 ± 29.0	17.6 ± 18.5	27.2 ± 27.7	-0.61 (-2.99, 1.76)	-5.94 (-7.99, -3.89)
White Grain	366 ± 170	362 ± 160	305 ± 118	58.4 (49.3, 67.5)	45.4 (36.1, 54.6)
Beans	28.1 ± 16.2	21.6 ± 12.2	23.0 ± 10.9	4.79 (3.58, 6.00)	-1.77 (-2.78, -0.77)
Fish	4.36 ± 3.80	3.86 ± 3.31	5.41 ± 2.98	-0.73 (-1.19, -0.27)	-1.23 (-1.68, -0.78)
Red Meat	17.2 ± 12.8	14.7 ± 11.8	23.5 ± 13.3	-5.99 (-7.02, -4.97)	-8.30 (-9.34, -7.27)
Processed Meat	5.10 ± 4.64	4.98 ± 4.73	3.66 ± 2.49	1.84 (0.84, 2.83)	2.13 (1.10, 3.16)
Chicken	20.0 ± 14.6	20.1 ± 14.6	23.4 ± 12.4	-4.03 (-5.11, -2.94)	-3.21 (-4.33,-2.10)
Egg	19.7 ± 10.9	18.7 ± 11.1	18.5 ± 10.2	1.29 (0.40, 2.17)	-0.09 (-1.00, 0.82)
Cheese	18.4 ± 10.3	11.7 ± 6.78	13.6 ± 7.05	4.27 (3.57, 4.96)	-0.34 (-0.97, 0.29)
Dairy	235 ± 135	218 ± 122	123 ± 72.2	103 (95.0, 112)	91.3 (83.4, 99.2)
Vegetables	448 ± 181	388 ± 156	268 ± 103	172 (161, 184)	108 (97.8, 119)
Juice	8.35 ± 6.77	6.78 ± 5.45	9.33 ± 3.50	-1.09 (-2.67, 0.49)	-2.16 (-3.76, -0.56)
Fruit	343 ± 201	311 ± 183	265 ± 114	69.5 (56.1, 82.9)	37.3(24.2, 50.4)
Dried Fruit	14.8 ± 12.4	13.8 ± 10.9	7.77 ± 5.73	6.77 (5.90, 7.65)	5.89 (5.03, 6.74)
Solid oil	11.9 ± 11.3	9.60 ± 9.16	8.56 ± 6.44	3.26 (2.55, 3.98)	1.08(0.50, 1.65)
Liquid Oil	6.01 ± 4.27	5.56 ± 4.05	4.93 ± 3.18	0.77 (0.44, 1.11)	0.39 (0.10, 0.67)
Olive/ Olive Oil	0.42 ± 0.42	0.48 ± 0.51	0.38 ± 0.17	0.18 (0.04, 0.33)	0.19 (-0.02, 0.40)
Nuts	5.90 ± 5.16	5.48 ± 4.15	4.17 ± 3.15	1.63 (1.28, 1.98)	1.21 (0.88, 1.54)
Sugar	28.8 ± 19.2	24.1 ± 16.0	19.6 ± 11.01	8.28 (7.19, 9.36)	3.85 (2.93, 4.76)
Sweets	17.2 ± 12.4	15.5 ± 10.6	19.8 ± 13.0	-2.93 (-4.00, -1.86)	-4.31 (-5.40, -3.23)
Sweet Drinks	38.0 ± 35.9	31.3 ± 30.8	35.3 ± 24.5	3.51 (0.38, 6.64)	10.8 (6.34, 15.17)
Tea	570 ± 303	559 ± 308	500 ± 233	72.1 (54.6, 89.7)	59.7 (42.7, 76.7)
Salt	4.25 ± 2.51	4.02 ± 2.16	2.68 ± 1.01	1.43 (1.27, 1.59)	1.17 (1.01, 1.32)

### Dietary patterns and the corresponding correlations

Through PCA of the food groups, we identified three major dietary patterns that were interpretable and comparable among the three datasets (Table 3). One pattern, characterized by high intakes of fruit, dairy, dried fruit, nuts, vegetables, olive/olive oil, and fish was named a ***Healthy*** pattern, while another, highly loaded with vegetables, legumes, white grain, liquid oils and very low intake of any protein sources (with the exception of legumes) was termed the ***Low Protein, High Carb*** pattern. The third pattern, included high intakes of white grain, processed meats, red meat, sugars and sweet drinks, and thus was named the ***Unhealthy*** pattern. These three patterns explained on average, 33.7%, 20.7%, and 16% of variance among the study participants, respectively.

**Table 3:** Factor loading matrix of 3 major dietary patterns identified in data gathered by the FFQ and 24 h

Food Group	Healthy Pattern			Low Protein, High Carb Pattern			Unhealthy Pattern		
	FFQ1	FFQ2	24 h	FFQ1	FFQ2	24 h	FFQ1	FFQ2	24 h
Whole Grain	0.17	0.34	0.16	-0.83	-0.22	-0.41	-0.39	-0.44	-0.69
White Grain	-0.75	-0.90	-0.65	0.53	0.28	0.50		0.17	0.30
Beans				0.16	0.13	0.29			
Fish	0.24	0.19	0.28						
Red Meat	0.15	0.22	0.31		-0.23		0.28		0.25
Processed Meat	0.12	0.21	0.24		-0.18		0.43	0.21	0.19
Chicken			0.27						
Egg						0.30	0.21	0.14	
Cheese	0.14	0.25	0.16	0.12		0.24			
Dairy	0.35	0.18	0.27		0.17			-0.30	
Vegetables	0.29	0.38		0.33	0.39	0.48	-0.15		-0.34
Juice	0.18	0.20	0.22				0.13	0.14	0.16
Fruit	0.46	0.50	0.46	0.36	0.16	0.25		0.20	
Dried Fruit	0.33	0.32	0.38				-0.13	-0.12	-0.11
Solid oil	-0.26	-0.21	-0.38	-0.13	-0.39	-0.40		-0.27	
Liquid Oil	0.18	0.22	0.45	0.22	0.28	0.40		0.30	
Olive/ Olive Oil	0.26	0.22	0.23	0.11	0.17				
Nuts	0.33	0.32	0.40						0.22
Sugar	-0.23	-0.13	-0.18	-0.18	-0.38	-0.21	0.47	0.17	0.22
Sweets	0.24	0.24	0.45		-0.33	-0.31	0.38	0.27	0.27
Sweet Drinks				-0.18	-0.29	-0.29	0.39	0.26	0.20
Tea				-0.15	-0.11	-0.13			
Salt							0.21	0.22	0.16

Values are factor loadings; absolute values < 0.1 are not displayed

Energy-adjusted and corrected SCC comparing FFQ1 vs. 24 h and FFQ2 vs. 24 h across the three identified patterns are shown in Table 4, ranging from 0.28 to 0.59 and 0.31 to 0.61, respectively. With the exception of the FFQ2 vs. 24 h in the Unhealthy pattern, all other correlations were above 0.5 and are considered to show acceptable validity. In comparing FFQ1 vs. FFQ2 for the reproducibility assessment (*n* = 891), correlations of 0.53 (Healthy), 0.57 (Low Protein, High Carb) and 0.34 (Unhealthy) were obtained (Table 4).

**Table 4:** Energy-adjusted and corrected Spearman correlation coefficients (SCC) and 95% CI for validity (FFQ1 and FFQ2 vs. 24 h) and reproducibility (FFQ1 vs. FFQ2) of the PERSIAN Cohort FFQ

	Energy-adjusted SCC (95% CI)			Corrected SCC (95% CI)		
	Healthy Pattern	Low Protein, High Carb Pattern	Unhealthy Pattern	Healthy Pattern	Low Protein, High Carb Pattern	Unhealthy Pattern
FFQ1 vs. 24 h	0.52 (0.47–0.57)	0.55 (0.50–0.59)	0.50 (0.45–0.55)	0.53 (0.48–0.57)	0.57 (0.52–0.61)	0.50 (0.45–0.55)
FFQ2 vs. 24 h	0.54 (0.49–0.59)	0.59 (0.54–0.63)	0.28 (0.22–0.34)	0.57 (0.52–0.61)	0.61 (0.57–0.65)	0.31 (0.25–0.37)
FFQ1 vs. FFQ2	0.53 (0.48–0.58)	0.57 (0.52–0.61)	0.34 (0.28–0.40)	-	-	-

### Agreement assessment by bland-altman plots

Bland-Altman plots assessing agreement between the FFQ and 24 h have been included as supplementary figures. Supplementary Fig. 2. shows the Bland-Altman plots for validity, and supplementary Fig. 3, the plots for reproducibility of the dietary pattern scores. Supplementary Tables 1 and 2 also present specific details about the plots, including the trend line, mean difference and 95% limits of agreement, indicating acceptable agreement between the questionnaires.

## Discussion

While nutrients were the main focus of nutrition epidemiology in the past, providing answers to the cause of malnutrition or food deficiencies, these nutrition disorders are less commonly seen today. Instead, parallel to urbanization and modernization of lifestyles in high income as well as low-to middle-income countries, a rise in NCDs is observed, with nutrition again playing an important, yet different role. Nutrition’s effects on NCD development does not pertain to single nutrients, but rather excessive or insufficient intake of various foods and the cumulative effects foods have on diseases over time; therefore, evaluating dietary patterns is an effective method to assess diet-NCD relationships [7, 20]. Dietary patterns reflect how individuals eat overtime and encompass a more holistic view of individuals’ dietary intake, taking into account combinations of foods consumed together as well as the synergistic interactions between food components that would be missed if single nutrients were studies [2, 3, 7, 21, 22].

Given that the PERSIAN Cohort Study, the largest multi-center cohort in Iran, investigates risk factors of NCDs, evaluating the validity and reproducibility of the FFQ used to gather dietary information of participants at the dietary pattern level was needed. We therefore, performed this study to validate the findings of future studies investigating diet-disease associations.

We identified 3 major dietary patterns, altogether explaining over 70% of variation in our study population. The corrected correlation coefficients (taking into account week-to-week variations in the 24 h), ranged from 0.31 to 0.61. The correlation coefficients pertaining to the first two patterns, accounting for over 50% of variation in the population, were above 0.5 in comparing FFQ1 and 2 to the 24 h. We therefore believe that our FFQ has acceptable validity in the identification of dietary patterns in the populations it is used in. In terms of reproducibility, energy-adjusted correlations ranged from 0.34 to 0.57, with again the first two patterns showing correlations above 0.5; therefore, our FFQ has acceptable reproducibility of findings as well.

While not many validation studies have performed dietary pattern analysis, those who have, reported validity correlations ranging from 0.29 to 0.66 (Mexican population), 0.5–0.85 (Swedish population), 0.32–0.63 (Japanese population), 0.48–0.74 (Iranian population), and 0.45–0.74 (American population) [1, 12, 23–25]. Most studies identified two major patterns, while some deriving three. The dietary patterns explained on average, 26% of the variance in the populations studied. For the reproducibility of the FFQs, correlations ranged from 0.55 to 0.8 in the abovementioned populations.

The first pattern we identified—termed as ***Healthy***—has many components similar to the Mediterranean diet, being positively loaded with fruits, dairy, nuts, olives/olive oil, and whole grains, while being the only pattern that included fish across all three questionnaires. This pattern is also negatively loaded for sugars, solid oils and white grains and can be compared to the healthy/prudent patterns observed in the American, Swedish and Japanese populations [1, 24, 25]. This pattern also shares similar components to the Iranian Traditional pattern identified by the TLGS study, being characterized by vegetables, eggs, red meat, fruit, dairy, whole grain and olive consumption [12]. While a comparison of this pattern to the socioeconomic status of participants was not made in this study, given the cost of these healthier foods in Iran, it is assumed that individuals with greater access and the more affluent individuals are following this healthier dietary pattern.

Our second pattern—the ***Low protein, High Carb***—had the highest load for white grains, which are used in most breads consumed in Iran. In addition, vegetables and liquid oils showed heavy loads in this pattern, with whole grains showing a strong negative load. Interestingly, almost all sources of protein were not seen to be consumed across the three questionnaires in this pattern. Only the intake of beans, as the fourth greatest load, was seen in FFQ1, 2 and the 24 h. Factor loads for the other major protein sources (red meat, chicken, eggs, dairy and processed meats) were either only present in one of the three questionnaires or were negative. This diet may be consumed by individuals of the lower income as high bread consumption is often seen in this population as a means for satiety, instead of protein sources. In addition, there is a high government subsidy for bread prices in Iran and they are easily obtained by the lower income population.

We kept the third pattern identified—the ***Unhealthy*** pattern—in our results as well, not because it was as strongly correlated as the other patterns, but because of the interesting components it contained, being high in sugar, processed meats, eggs, juice, and the only pattern including salt as a factor, while at the same time being negatively loaded with whole grains, dried fruits, and vegetables. While the main component of a Western diet, including high fat consumption is not seen in this pattern, it is a clear unhealthy diet and we believed that it was valuable to be shown as a finding. The correlations between FFQ1 and the 24 h were 0.5, but lower when FFQ2 was compared to the 24 h (0.31 and 0.28 for corrected and uncorrected, respectively).

Differences were noted in the factor loadings of foods in the various patterns, which could be explained by true changes in individuals’ dietary intake over the 1-year study period, random statistical variations, as well as differences in the data collection methods used in the FFQ and the 24 h [1, 24]. The 24 h were chosen to be completed in this study as the most applicable reference method due to participants’ low literacy levels, and while we did obtain two 24 h monthly for 12 months to capture greater variability in individuals’ dietary intake, it is still not unreasonable to believe that by chance, certain foods may not have been reflected at all or as much as they are truly consumed by an individual, in their 24 h, but were reported in their FFQ. In contrast, while both questionnaires rely on memory and may be affected by errors in recall, reporting foods consumed in the previous 24 h has fewer errors than recalling typical intake over the year prior, and therefore, over/underestimation of intake by the FFQ in comparison to the 24 h is expected, affecting the factor loads of foods in the patterns obtained. Despite these differences however, the overall components of the patterns were similar and correlations obtained in our study show suitability of the PERSIAN Cohort FFQ in assessing dietary patterns compared to 24 h.

One of the strengths of our study is its diverse and multi-center population, encompassing individuals from all major ethnicities of Iran, as compared to the participants of the previously validated FFQ in Iran, that included participants from one ethnic population [11], the elderly [26], a specific disease population [27], or those residing in Iran’s capital city, whose dietary habits are highly affected by urbanization and modern lifestyles [12].

Another strength is that we designed an FFQ that is shorter than those previously validated, which can be more efficient for the investigators administering this questionnaire, and less tiring for the participants responding to it. While the validity and reproducibility correlations obtained by some of the other studies are in some cases higher than those obtained by us, this was expected and was a trade-off, as our FFQ was designed to be more general yet concise, and only foods that were believed to be consumed regularly throughout Iran were included, leaving out specific foods that may have been part of our participants’ diets in specific areas of Iran, but not others. On the other hand, however, our FFQ was able to incorporate the local dietary habits of our diverse population, in order to ensure that no major energy-contributing foods or foods with specific nutrients that are different from others included in the questionnaire, are disregarded. These local foods were equated to the main food items on the FFQ, based on their major nutrients.

The PERSIAN Cohort FFQ was interviewer-administered because a considerable proportion of the cohort population had low literacy levels and we wanted to ensure that all FFQs are completed with the same level of accuracy. All interviewers were trained by the same individual and used the same tools to complete the FFQs as well as the 24 h, in order to limit biases and measurement errors. But this may have at the same time increased the correlated errors in our findings, inflating the validation results obtained. Another fact that may have affected our results due to correlated errors, is the use of 24 h as the reference method, since it shares two major errors (relying on memory and estimation of portion sizes) with the FFQ, in comparison to diet records that do not. Completing diet records however, requires high motivation and literacy levels and again, since over 40% of our population had limited education, use of the 24 h was the most suitable alternative in our study. Examining objective reference methods is another way to overcome this limitation as they contain no correlated errors with the FFQ; this can be performed in future studies.

The subjective food grouping used in this study is another limitation, as different grouping of the FFQ food items would have yielded different results; albeit this limitation is shared by all validation studies and no defined food groups are used by all to assess food group or dietary pattern validity/reproducibility [20]. The decision regarding the number of patterns identified, as well as the food items chosen to characterize each pattern are also subjective and could be interpreted differently by other researchers.

## Conclusions

Given the fact that studying dietary patterns can provide us with a better understanding of nutrition’s role in the etiology of the most common NCDs, validity and reproducibility of the PERSIAN Cohort FFQ was evaluated and the questionnaire was found to be suitable for assessing the dietary patterns of the Iranian population with diverse ethnic backgrounds and dietary habits.

### Electronic supplementary material

Below is the link to the electronic supplementary material.

Supplementary Material 1

## Data Availability

Data as well as analytical codes supporting the conclusions of this article will be made available by the authors, upon request.

## Abbreviations

FFQ: Food frequency questionnaire
PERSIAN: Prospective Epidemiological Research Studies in IrAN
24HR: 24-hour dietary recalls
SCC: Spearman’s correlation coefficients
NCD: Non-communicable Diseases
GCS: Golestan Cohort Study
TLGS: Tehran Lipid and Glucose Study
USDA: United States Department of Agriculture
