# Supplementary material for: Validity and reproducibility of the PERSIAN Cohort food frequency questionnaire: assessment of major dietary patterns
Source: Nutr J. 2024 Mar 13;23:35. doi: 10.1186/s12937-024-00938-0 (PMC10935787; doi:10.1186/s12937-024-00938-0)
Supplement: Supplementary file 1 — Supplementary Material 1 [file 12937_2024_938_MOESM1_ESM.docx]

**Supplementary Materials:**

**Validity and reproducibility of the PERSIAN Cohort food frequency questionnaire: assessment of major dietary patterns**

Sareh Eghtesad, Sahar Masoudi, Maryam Sharafkhah, Bahram Rashidkhani, Ali Esmaeili-Nadimi, Farid Najafi, Elnaz Faramarzi, Reza Homayounfar, Pedram Ebrahimnejad, Alireza Ansari-Moghaddam, Mohammadreza Mirjalili, Hossein Poustchi, Walter Willett, Reza Malekzadeh, Azita Hekmatdoost

**Supplementary Figure 1. Scree Plots of the PCA model**


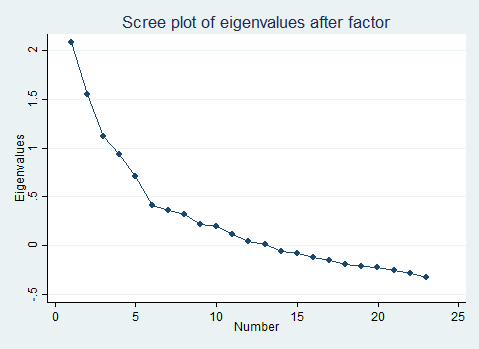


**A**


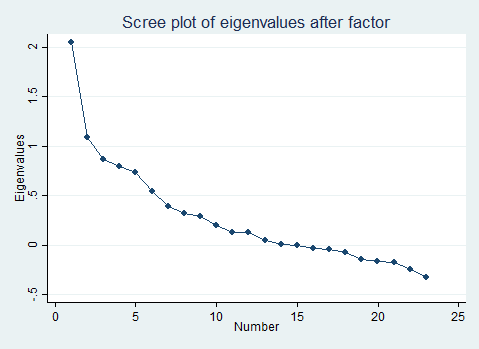

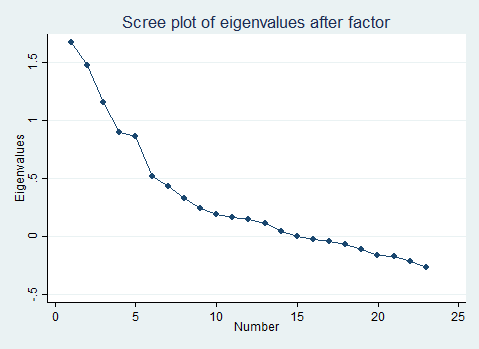


**B**

**C**

**Supplementary Figure 1. Scree Plots of the PCA model:** A – Scree plot of the 24HRs, B – Scree Plot of FFQ1 and C – Scree Plot of FFQ2

**Supplementary Figure 2. Bland-Altman Plots assessing agreement between the dietary pattern scores in the validation analysis**


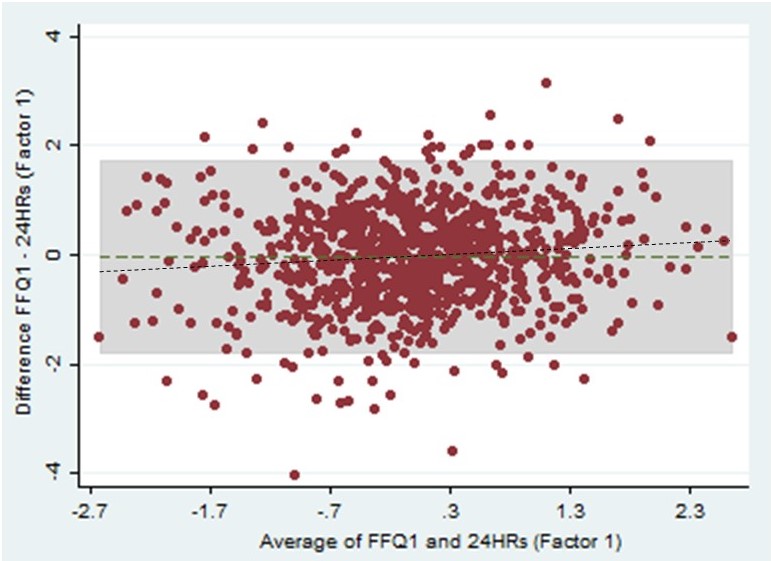

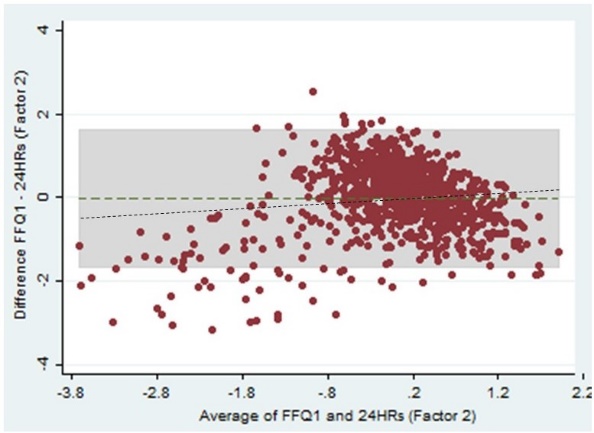


**B**


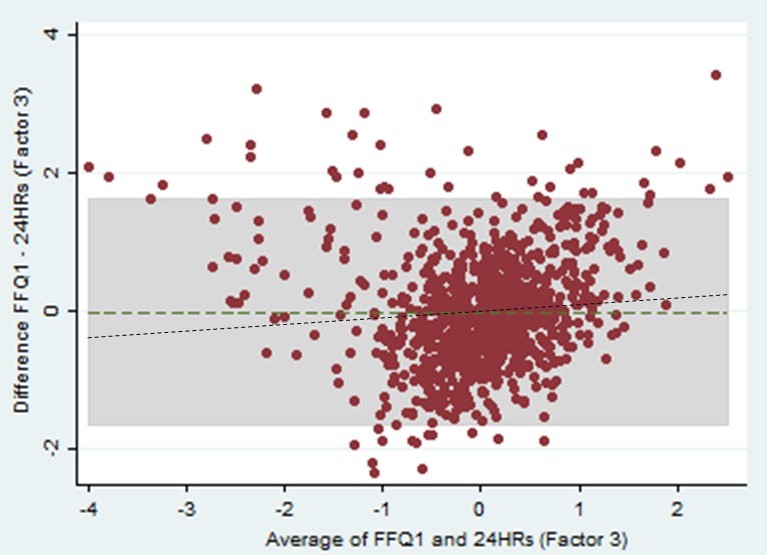


**C**


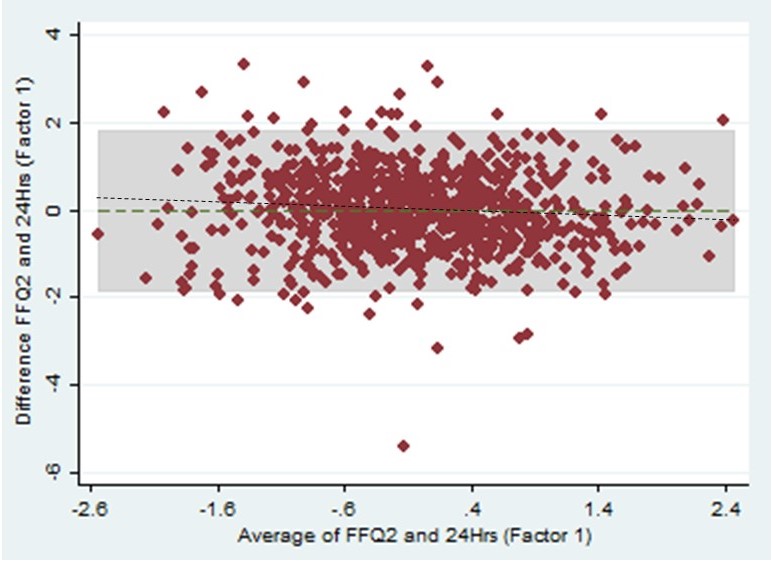


**D**


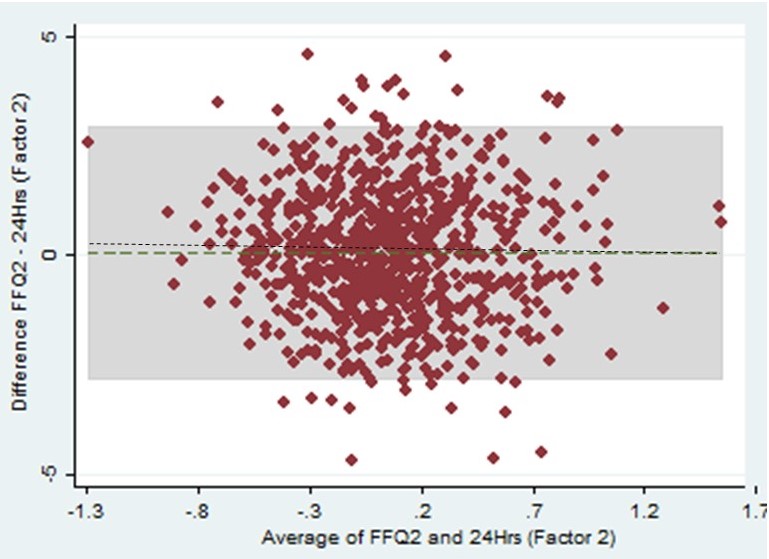


**E**


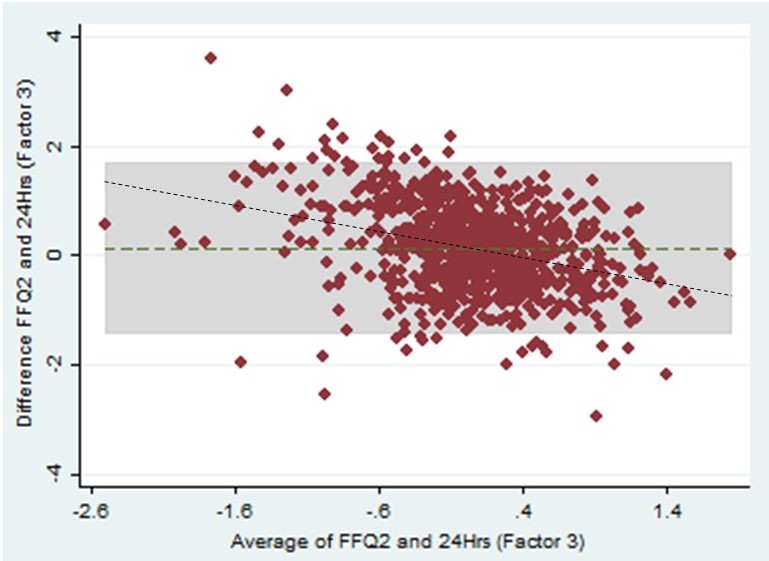


**F**

**A**

| **Supplementary Table 1.** | **Trend Line** | **Mean Difference** | **95% Limits of Agreement** |
| --- | --- | --- | --- |
| 1. FFQ1 and 24HRs Factor 1: Healthy Pattern | -0.02 + 0.11*Average | -0.02 | -1.78 , 1.73 |
| 1. FFQ1 and 24HRs Factor 2: Low Protein, High Carb Pattern | -0.02+ 0.12*Average | -0.02 | -1.6 , 1.62 |
| 1. FFQ1 and 24HRs Factor 3: Unhealthy Pattern | -0.01 + 0.09*Average | -0.01 | -1.63 , 1.62 |
| 1. FFQ2 and 24HRs Factor 1: Healthy Pattern | -0.02 + (-0.09)*Average | -0.02 | -1.83 , 1.79 |
| 1. FFQ2 and 24HRs Factor 2: Low Protein, High Carb Pattern | 0.08+ (-0.11)*Average | .07 | -2.79 , 2.94 |
| 1. FFQ2 and 24HRs Factor 3: Unhealthy Pattern | 0.17+ (-0.53)*Average | 0.137 | -1.42 , 1.69 |

**Supplementary Figure 2 and Table 1. Bland-Altman Plots assessing agreement between the dietary pattern scores in the validation analysis:** FFQ1 vs. 24HRs agreements are shown in plots A-C as: Healthy (A), Low Protein, High Carb (B), and Unhealthy (C), while the FFQ2 vs. 24HRs plots are shown in D-E as: Healthy (D), Low Protein, High Carb (E), and Unhealthy (F). The dashed line represents mean difference while the shaded region shows the limits of agreement (±2 SD from the mean difference). The dotted line represents the trend line. Specific information on the trend line, mean differences and 95% limits of agreement are shown in supplementary table 1.

**Supplementary Figure 3. Bland-Altman Plots assessing agreement between the dietary pattern scores in the reproducibility analysis**


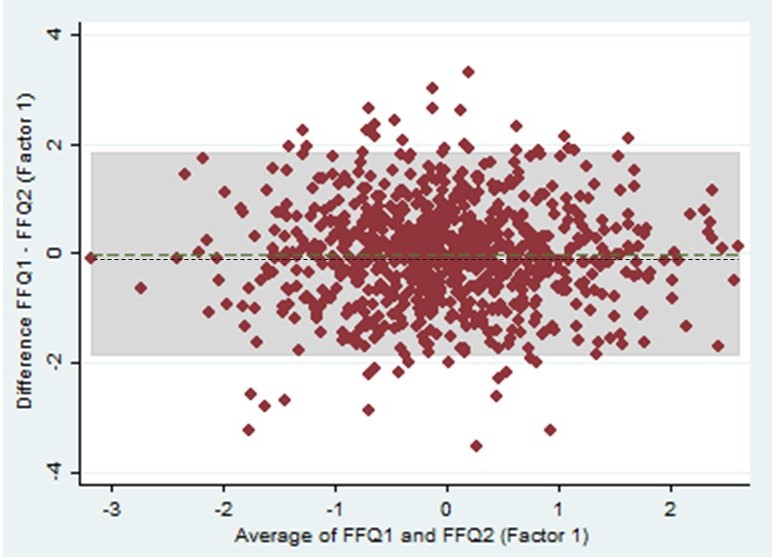

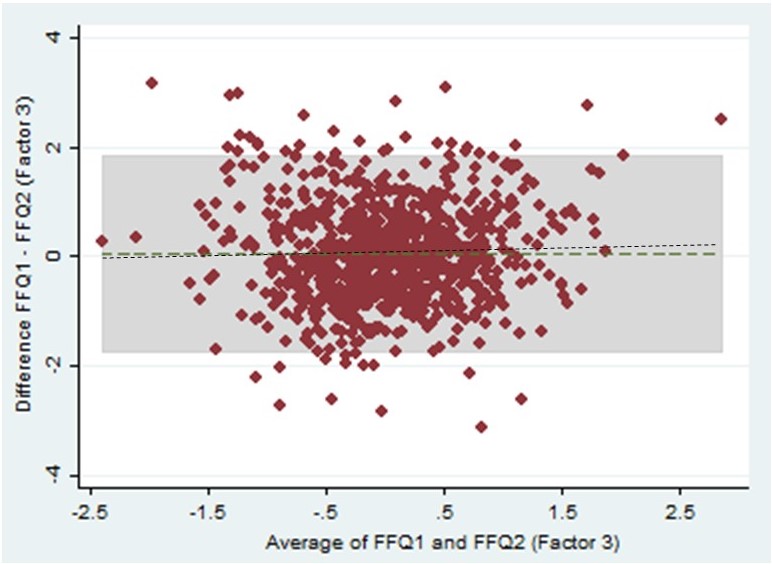

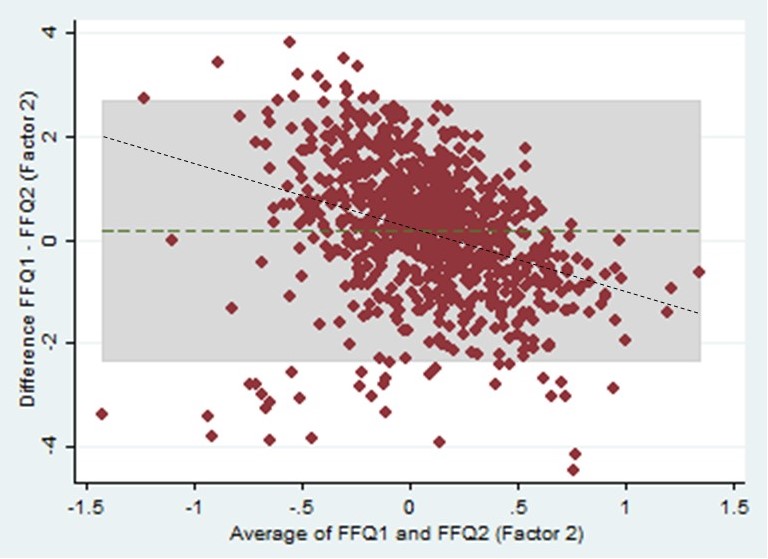


**C**

**B**

**A**

| **Supplementary Table 2.** | **Trend Line** | **Mean Difference** | **95% Limits of Agreement** |
| --- | --- | --- | --- |
| 1. FFQ1 and FFQ2 Factor 1: Healthy Pattern | -0.02 + 0.00*Average | -0.016 | -1.86 , 1.82 |
| 1. FFQ1 and FFQ2 Factor 2: Low Protein, High Carb Pattern | 0.27 + (-1.20)*Average | 0.17 | -2.32 , 2.67 |
| 1. FFQ1 and FFQ2 Factor 3: Unhealthy Pattern | 0.05 + 0.06*Average | 0.05 | -1.72 , 1.8 |

**Supplementary Figure 3 and Table 2. Bland-Altman Plots assessing agreement between the dietary pattern scores in the reproducibility analysis:** FFQ1 vs. FFQ2 agreements are shown in plots A-C as: Healthy (A), Low Protein, High Carb (B), and Unhealthy (C). The dashed line represents mean difference while the shaded region shows the limits of agreement (±2 SD from the mean difference). The dotted line represents the trend line. Specific information on the trend line, mean differences and 95% limits of agreement are shown in supplementary table 2.
